# Atypical Takayasu Arteritis Presenting as Ischemic Stroke in a Young Female Despite Normal ESR and Preserved Peripheral Pulses: A Case Report

**DOI:** 10.7759/cureus.114610

**Published:** 2026-08-16

**Authors:** Anuradha Gupta, Navjot Singh, Kashish Narula, Karan Goyal

**Affiliations:** 1 Research, Government Medical College and Hospital, Nagpur, Nagpur, IND; 2 Internal Medicine, Maharishi Markandeshwar Institute of Medical Sciences and Research, Ambala, IND; 3 Research, Punjab Institutes of Medical Sciences, Jalandhar, IND; 4 Internal Medicine, Government Medical College, Amritsar, Amritsar, IND; 5 Radiology, Maharishi Markandeshwar Institute of Medical Sciences and Research, Ambala, IND

**Keywords:** atypical vasculitis, carotid artery stenosis, cerebrovascular accident (stroke), inflammatory biomarkers, ischemic stroke, large vessel vasculitis, rheumatology disorder, supraclinoid internal carotid artery, takayasu arteritis (tak), young age stroke

## Abstract

Takayasu's arteritis (TA) is a chronic granulomatous vasculitis that primarily affects young women and involves the aorta and its major branches. Although neurological manifestations, such as stroke (15.8% prevalence in TA), are uncommon, they can be debilitating. We present the case of a 30-year-old woman from Haryana, India, who presented to the hospital with acute right-sided hemiparesis and dysphasia. Despite normal inflammatory markers, neuroimaging revealed chronic infarcts accompanied by significant stenosis of the intracranial arteries and extensive collateral formation. A computed tomographic angiography (CTA) showed diffuse circumferential thickening and severe stenosis of the right internal carotid artery and its branches, indicative of large-vessel vasculitis. Notably, the involvement of the intracranial internal carotid artery represented an uncommon manifestation of TA, as the disease predominantly affects the extracranial aorta and its major branches, with intracranial arterial involvement being relatively rare. Based on clinical findings, radiological evidence, and diagnostic criteria, a diagnosis of TA was established. Based on early vascular imaging, the patient was treated with corticosteroids, methotrexate, and antiplatelet therapy, which led to significant clinical improvement. This case underscores the importance of considering vasculitic causes like TA in young stroke patients, especially when conventional risk factors are absent.

## Introduction

Takayasu’s arteritis (TA) is a chronic inflammatory disease of large blood vessels that mainly affects the aorta and its major branches, including the brachiocephalic, carotid, subclavian, vertebral, and renal arteries, and can also involve the coronary and pulmonary arteries [1]. It results in a range of ischemic symptoms caused by the narrowing of the vessels or the formation of thrombi [1]. Nearly all patients develop ischemic complications in the affected vessels, presenting with symptoms such as dizziness, syncope, visual disturbances, diminished or absent pulses, and differences in systolic blood pressure between the arms [1]. Stroke plays a key role in causing functional impairment in individuals with TA [2]. Studies suggest that 10-20% of patients with TA experience stroke, with pooled data indicating a prevalence of about 15.8% [3]. At the time of confirmed diagnosis of TA, significantly elevated erythrocyte sedimentation rate (ESR) levels were commonly observed in younger patients [4].

This case report describes a young female patient from Haryana, India, who presented with right-sided hemiplegia, with findings that were not consistent with the initial non-contrast computed tomography (NCCT) scan of the brain. Her inflammatory markers and autoimmune profile were within normal limits. Clinical examination demonstrated well-palpable peripheral pulses with no discrepancy in systolic blood pressure between the arms. Despite the absence of typical clinical features, a strong index of suspicion prompted further radiological evaluation, which ultimately confirmed a diagnosis of TA with an atypical presentation. This case underscores the importance of recognizing the imaging features of TA and considering vasculitic etiologies when evaluating acute stroke in young patients. While ischemic stroke in active inflammatory disease may result from ongoing vascular inflammation and thromboembolic events, chronic stenotic disease is more commonly associated with progressive arterial narrowing and cerebral hypoperfusion. The clinical and radiological findings in our patient, including normal inflammatory markers, chronic infarcts, severe arterial stenosis, and extensive collateral formation, were more suggestive of the latter.

## Case presentation

A 30-year-old Indian woman presented with weakness of the right upper and lower limbs for six days and difficulty swallowing for one day. She also reported intermittent pain and numbness in her upper limb for the past three to four months. There was no history of trauma to the affected limb or neck, cervical pain radiating to the upper limb, neck movements precipitating the symptoms, Raynaud’s phenomenon, digital color changes, or joint swelling. There was no history of connective tissue disease, diabetes mellitus, smoking, or previous thromboembolic events. The absence of these features made musculoskeletal, cervical radiculopathy, peripheral neuropathy, vasospastic, and atherosclerotic causes less likely, prompting further evaluation for a large-vessel vasculitis. She had no history of fever, head injury, or seizure. There was no significant past medical or surgical history, nor was she on any long-term medications. The patient also denied use of hormonal therapy, oral contraceptives, steroid intake, and traditional desi medicine. There was no known family history of autoimmune, cardiac, or early-onset cerebrovascular diseases. 

She denied current or past use of alcohol, tobacco, or any recreational drugs. She had a history of two full-term normal vaginal deliveries and one spontaneous abortion following a traumatic fall from a motorcycle that resulted in per-vaginal bleeding. In the emergency room, her Glasgow Coma Scale (GCS) score was 14/15. On physical examination, she was conscious and cooperative. She was well oriented to time, place, and person, and was also following verbal commands. Her pulse rate was 104 beats per minute in the left radial artery, regular, normal character, normal volume, all peripheral pulses palpable, and no vessel wall thickening was noted. Her blood pressure was 104/70 mmHg measured in the right arm in the supine position, 106/74 mmHg measured in the left arm in the supine position, 108/74 mmHg in the left lower limb, and 106/76 mmHg in the right lower limb. Her respiratory rate was 16 breaths per minute and was thoraco-abdominal type; oxygen saturation was 98% on room air; and temperature was 98°F measured in the left axilla. On clinical examination, mild pallor was present. No cyanosis, clubbing, icterus, oedema, or lymphadenopathy was noted.

On central nervous system examination, she was well oriented to time, place, and person. Her immediate, recent, and remote memory was intact. Her attention span, language, and speech were also normal. She scored 28/30 on the Mini-Mental Status Examination. A detailed cranial nerve (CN) examination was performed. The olfactory nerve/CN I was intact, with no reported abnormality of smell. The optic nerve/CN II examination was normal, with preserved visual acuity, visual fields, and pupillary light responses. The oculomotor, trochlear, and abducens nerves/CN III, IV, and VI were intact, with normal extraocular movements, equal and reactive pupils, and no ptosis, diplopia, or nystagmus. The trigeminal nerve/CN V was intact, with normal facial sensation and preserved movements of the muscles of mastication. The facial nerve/CN VII examination revealed symmetrical facial movements without facial weakness. The vestibulocochlear nerve/CN VIII was intact, with no apparent hearing abnormality. The patient reported difficulty in swallowing for one day, but examination of the glossopharyngeal and vagus nerves/CN IX and X did not reveal any other clinically evident abnormality; there was no documented palatal asymmetry or voice abnormality. The accessory nerve/CN XI was intact, with normal shoulder shrug and head rotation against resistance. The hypoglossal nerve/CN XII was intact, with normal tongue movements and no deviation or fasciculations. Overall, the cranial nerve examination was unremarkable apart from the patient's reported symptom of dysphagia, with no other focal cranial nerve deficits identified.

On motor examination, muscle bulk was preserved, with no focal muscle wasting or fasciculations. Muscle tone was otherwise unremarkable. Power was grade 3/5 in the right upper and lower limbs, while power was preserved on the left side. There was no other focal motor deficit. The findings were consistent with a right-sided hemiparesis. Deep tendon reflexes were assessed bilaterally. Reflexes were otherwise symmetrical, except for a positive right plantar response/Babinski sign. No other pathological reflexes were elicited. Sensory examination was performed for superficial and deep sensations. Touch, pain, temperature, vibration, and joint-position sense were preserved, with no definite sensory level or focal sensory deficit identified. There was no evidence of cortical sensory impairment. Coordination was assessed using appropriate bedside cerebellar tests. Finger-to-nose and heel-to-shin testing were normal, with no dysmetria or intention tremor. There was no clinical evidence of cerebellar dysfunction. Gait could not be assessed adequately at initial presentation because of the patient's right-sided hemiparesis. Following neurological improvement during the hospital course and subsequent follow-up, the patient was able to ambulate with a normal gait, without an evident residual gait abnormality. The rest of her systemic examinations were unremarkable.

Vascular system examination revealed no skin color changes, trophic changes, ulcers, gangrene, scars, dilated superficial veins, or signs of limb ischemia on inspection. All peripheral pulses, which included radial, ulnar, brachial, carotid, femoral, popliteal, posterior tibial, and dorsalis pedis, were symmetrically palpable. No radial-radial delay, radio-femoral delay, or difference in pulse volume between limbs was appreciated. Right-sided carotid bruits were present. No engorged neck veins were seen, and jugular venous pressure was not raised. No significant inter-arm blood pressure difference was noted as specified earlier in physical examination findings. The capillary refill time was one second. No hair loss, shiny or thin skin, muscle wasting, or brittle nails were seen. Temperature and color were normal in all four limbs. No swelling, changes in muscle bulk, skin changes, ulcers, or peripheral ischemic changes were noted in all four limbs.

Her National Institutes of Health Stroke Scale (NIHSS) score was 5, and modified Rankin Scale score was 4 at presentation. Laboratory investigations showed mild anemia and normal inflammatory markers. Although inflammatory markers are frequently elevated in active TA, they may remain within normal limits in patients with chronic or less active disease despite persistent vascular remodeling and fixed arterial stenosis. Antinuclear antibody (ANA) testing by indirect immunofluorescence assay (IFA) was negative.

The baseline investigation reports are illustrated in Table 1.

**Table 1:** Baseline laboratory reports of the patient during admission SGOT: serum glutamic-oxaloacetic transaminase, SGPT: serum glutamine-pyruvic transaminase, ALP: alkaline phosphatase, CRP: C-reactive protein, ESR: erythrocyte sedimentation rate, ANA: antinuclear antibody, IFA: indirect immunofluorescence assay

Laboratory parameters	Values	Normal value
Haemoglobin	10.2 gm%	12-15 gm%
Total leucocyte count	6800 cell/mm^3^	4000-10000 cell/mm^3^
Platelet count	283000 cell/mm^3^	150000-400000 cell/mm^3^
Urea	22 mg/dl	18-40 mg/dl
Creatinine	0.63 mg/dl	0.7-1.2 mg/dl
Uric acid	6.0 mg/dl	2-5 mg/dl
Sodium	137 mEq/L	135-145 mEq/L
Potassium	4.4 mEq/L	3.5-5.5 mEq/L
Chloride	102 mEq/L	96-106 mEq/L
Bilirubin total/direct	0.57/0.14 mg/dl	0.2-1.0/0.0-0.2 mg/dl
SGOT/SGPT	21 U/L/10U/L	5-35 U/L
ALP	93 U/L	28-112 U/L
Total protein	6.7 gm/dl	6-8 gm/dl
Albumin	4.1 gm/dl	3.5-5.3 gm/dl
ANA by IFA	Negative	<10.00 IU/ml
ESR	10 mm at the first hour	0-20 mm at the firsthour
CRP	6.3 mg/dl	0-6 mg/dl

Electrocardiogram, chest X-ray, abdominal ultrasound, and echocardiography were normal. The NCCT head was suggestive of an ill-defined hypodensity seen in the right cerebral semi-ovale, likely a chronic infarct.

The patient was admitted with a provisional diagnosis of left-sided ischemic stroke. 

**Figure 1:**
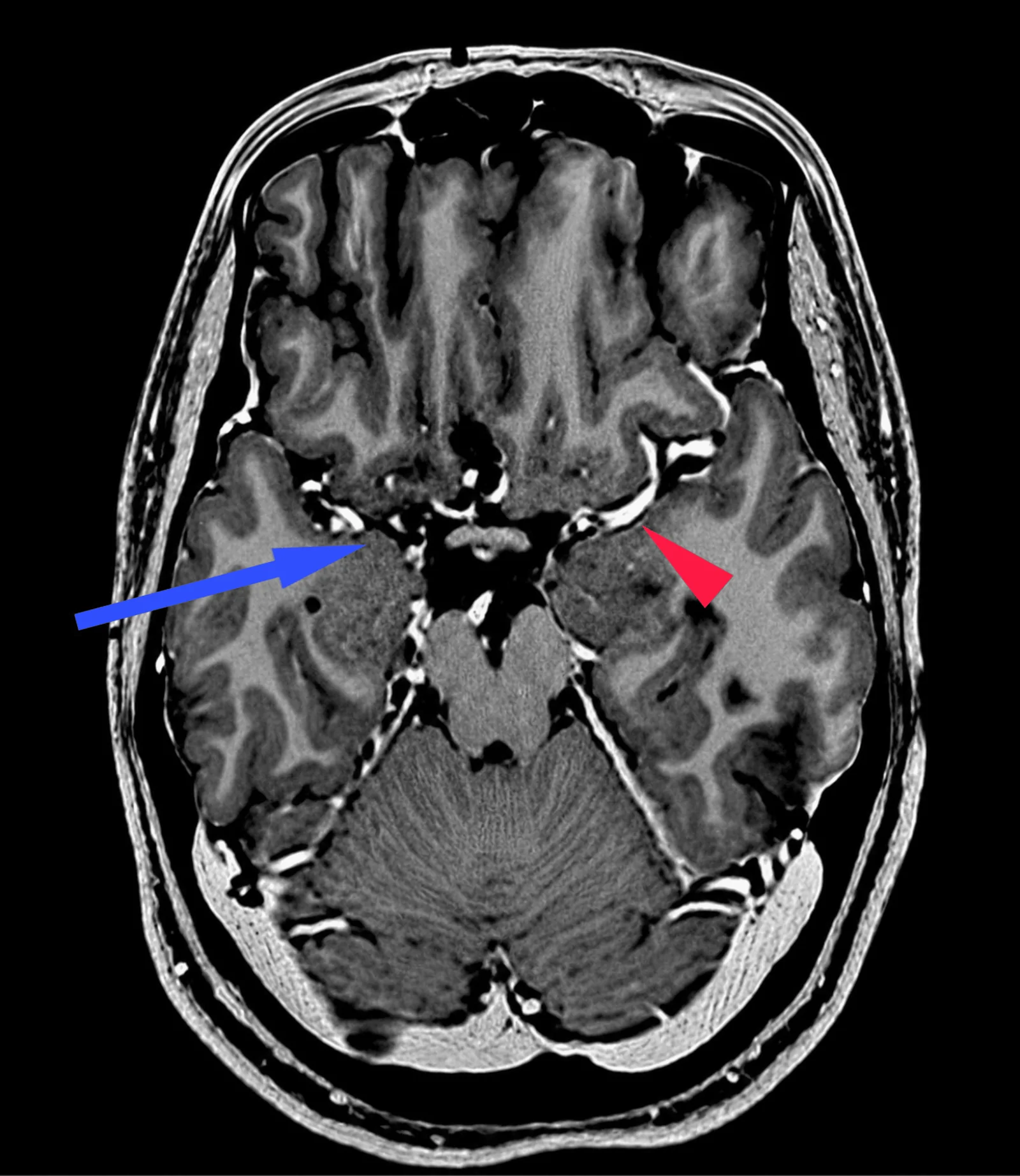
Contrast-enhanced cerebral magnetic resonance imaging of the patient Narrowing of the communicating part of the right internal carotid artery with collaterals (blue arrow) and normal findings on the left side (red arrow-head).

**Figure 2:**
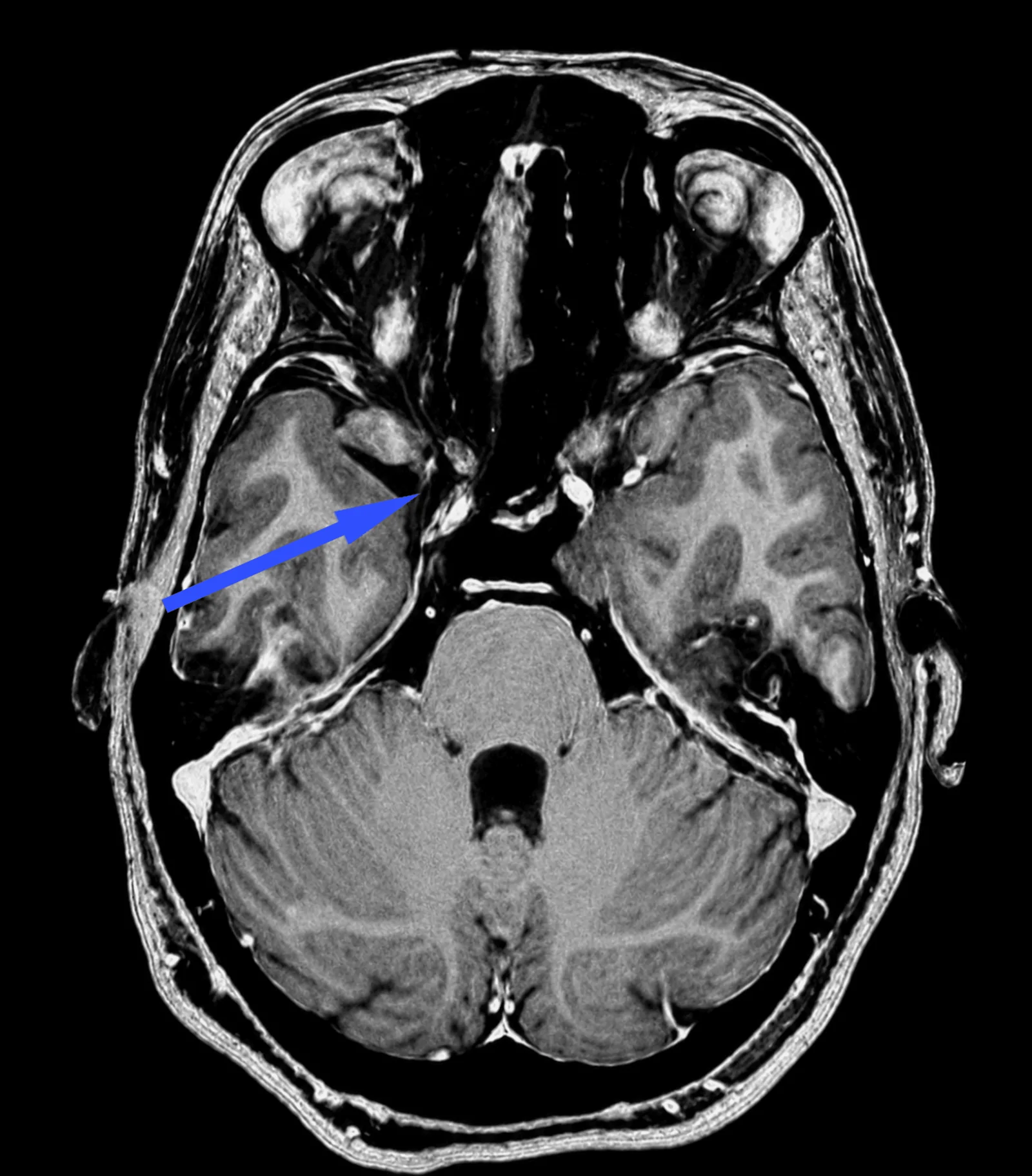
Contrast-enhanced cerebral magnetic resonance imaging of the patient. Abrupt narrowing in the P1 segment of the right posterior cerebral artery (blue arrow) with multiple collaterals along its course.

As the findings on the initial NCCT head scan did not adequately explain the patient's acute neurological deficits, a contrast-enhanced cerebral magnetic resonance (MRI) imaging scan was performed for further characterization of the cerebral lesions and assessment of the intracranial vasculature. The clinicoradiological discrepancy raised suspicion of an underlying vascular pathology. The contrast-enhanced MRI scan showed focal gliotic and encephalomalacic changes in the right parietal lobe, likely sequelae of a chronic infarct, with a few chronic lacunar infarcts within the white matter of the right frontal lobe. There was evidence of abrupt narrowing in the right supraclinoid internal carotid artery (Figure 1) and the P1 segment of the right posterior cerebral artery with multiple collaterals (Figure 2) along the course, replacing them with the right middle cerebral artery, right anterior cerebral artery, and right posterior cerebral artery, likely receiving supply from the opposite side.

**Figure 3:**
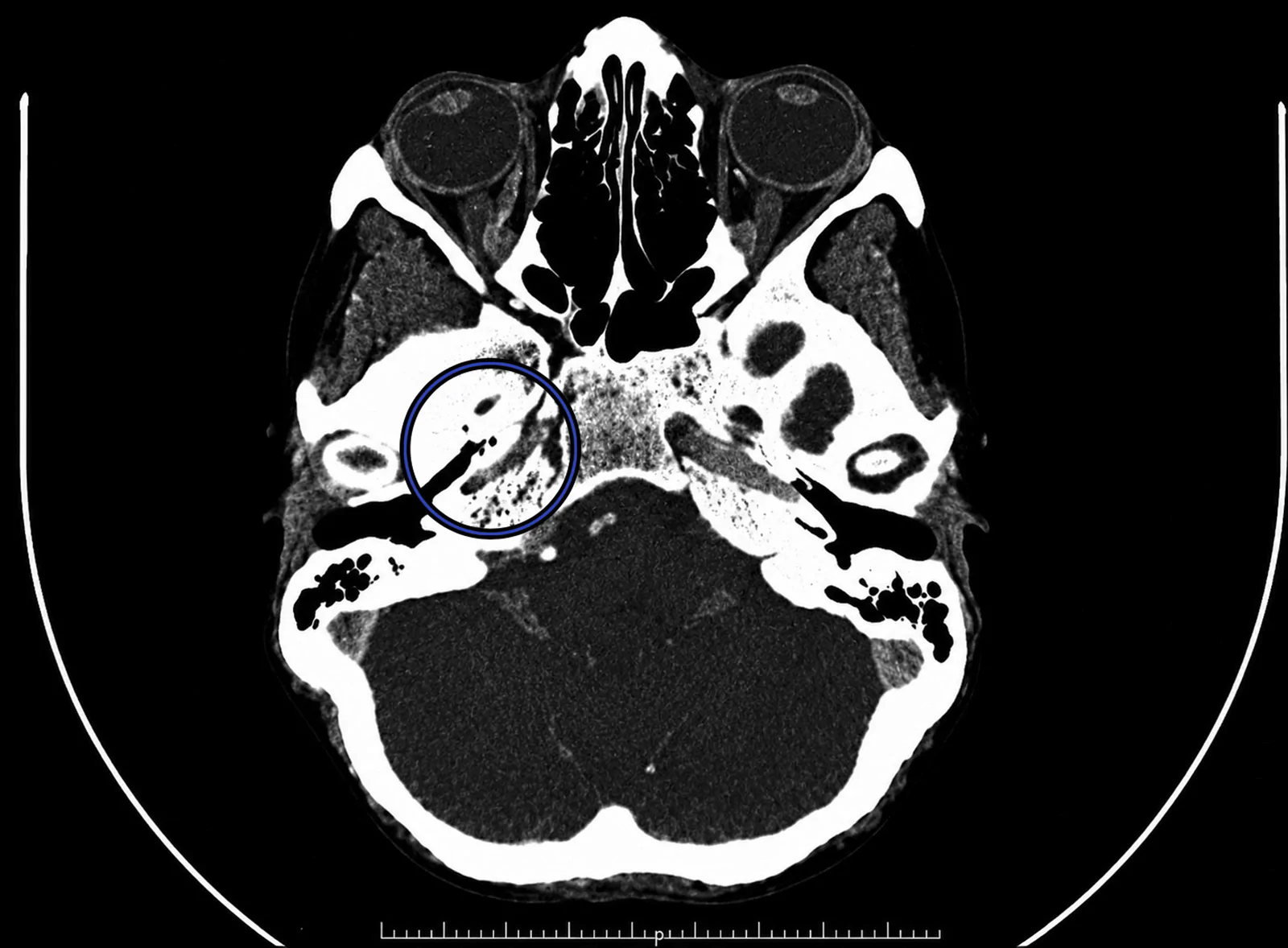
Carotid and cerebral computed tomographic angiography The right internal carotid artery diffusely narrowed (blue circle) in its intracranial course in the petrous segment.

**Figure 4:**
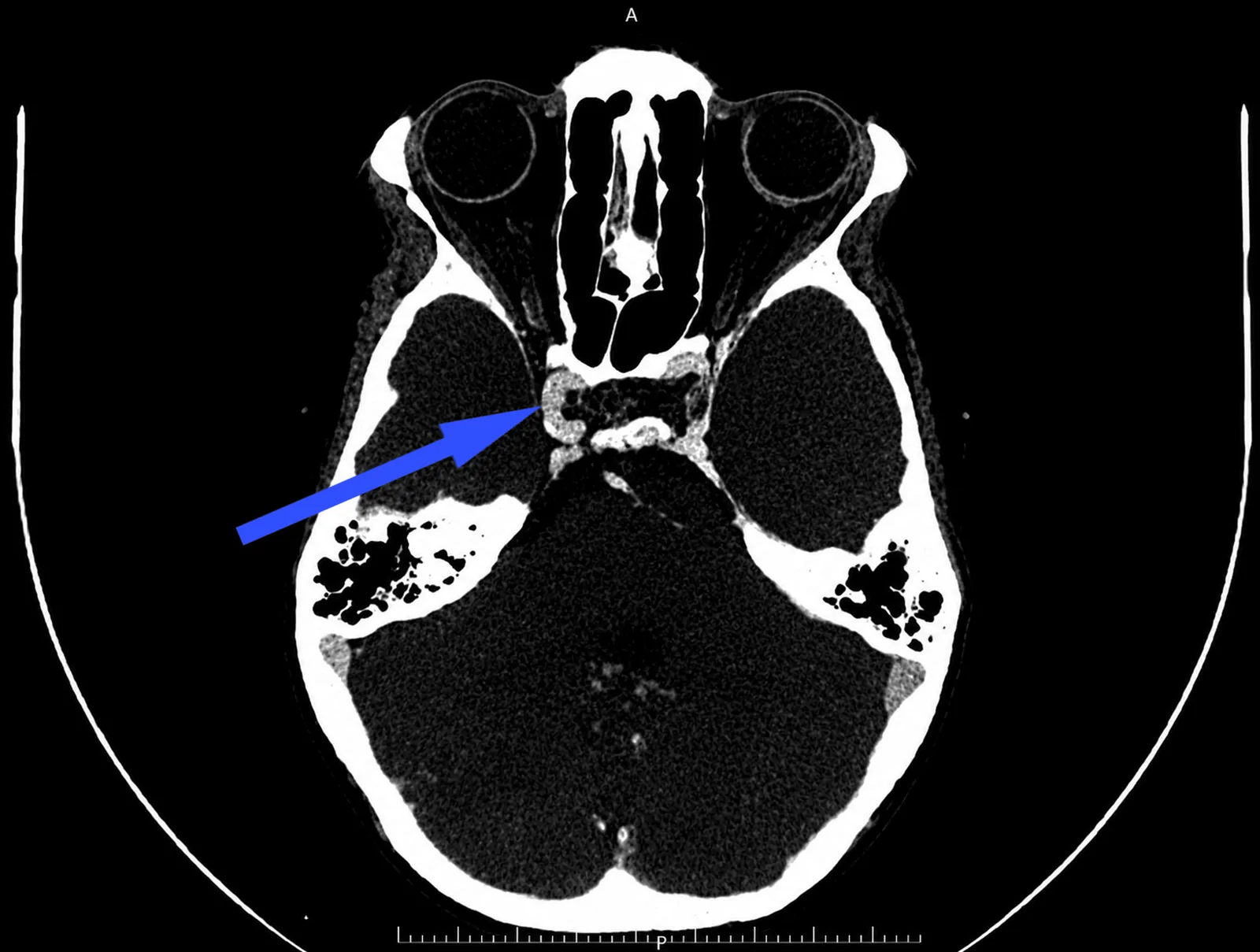
Carotid and cerebral computed tomographic angiography Thick-walled calcification in the cavernous segment of the right internal carotid artery (blue arrow).

**Figure 5:**
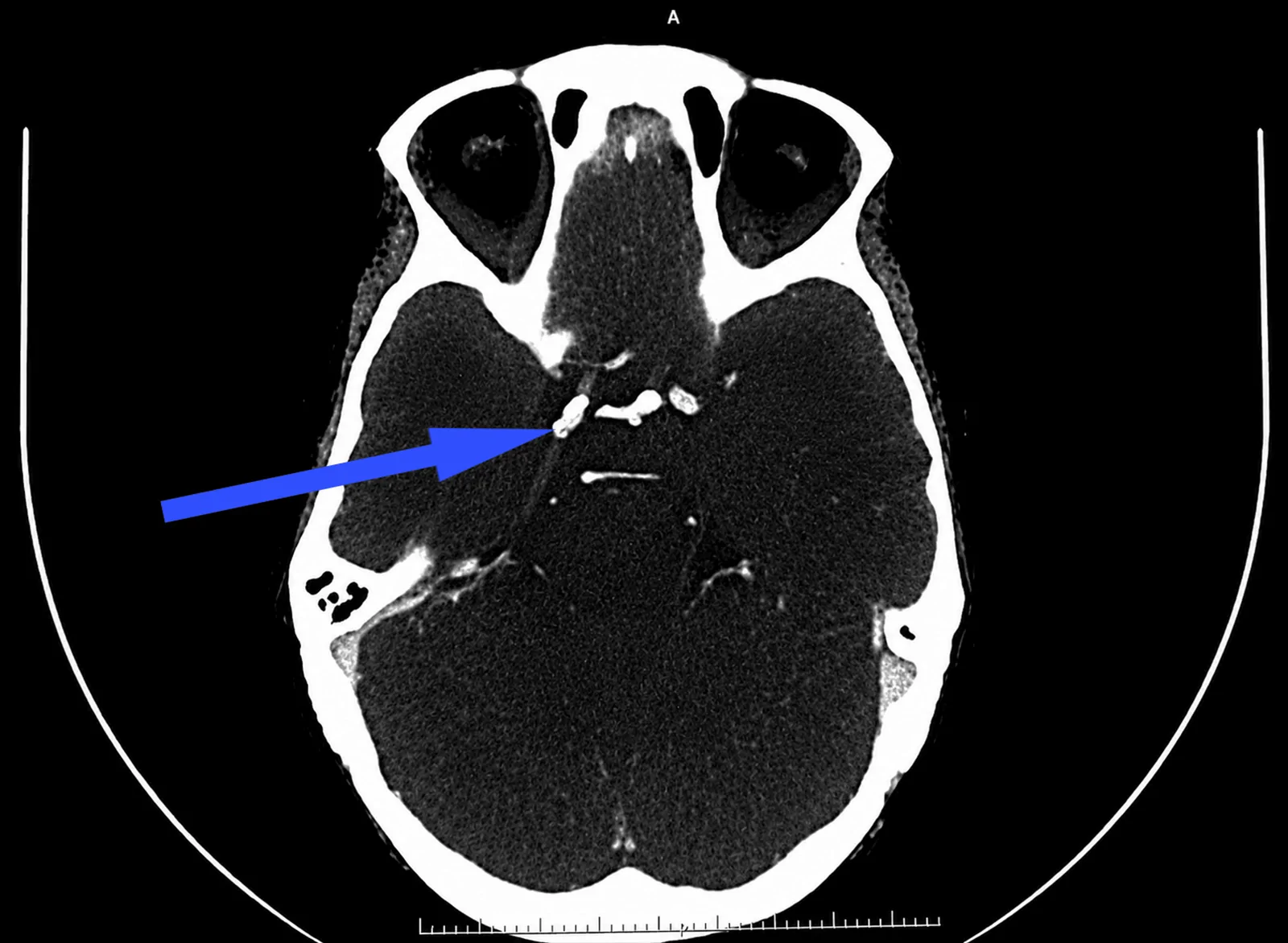
Carotid and cerebral computed tomographic angiography Thick eccentric wall calcification with severe stenosis in the right supraclinoid segment (blue arrow).

**Figure 6:**
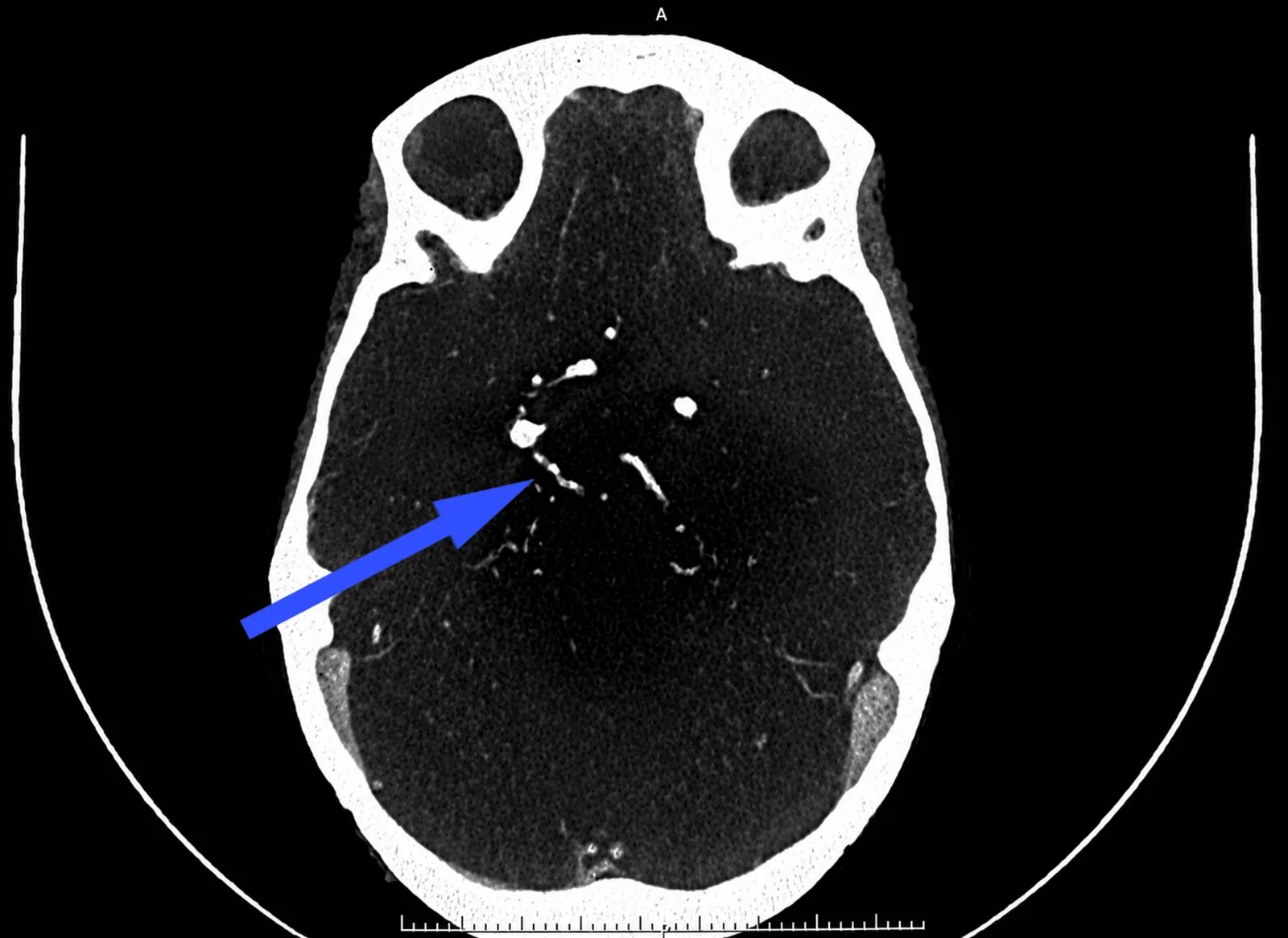
Carotid and cerebral computed tomographic angiography The P1 segment of the right posterior cerebral artery is hypoplastic, and the P2 segment is seen as a continuation of the right posterior communicating artery ( blue arrow), suggestive of fetal origin of the right posterior cerebral artery.

**Figure 7:**
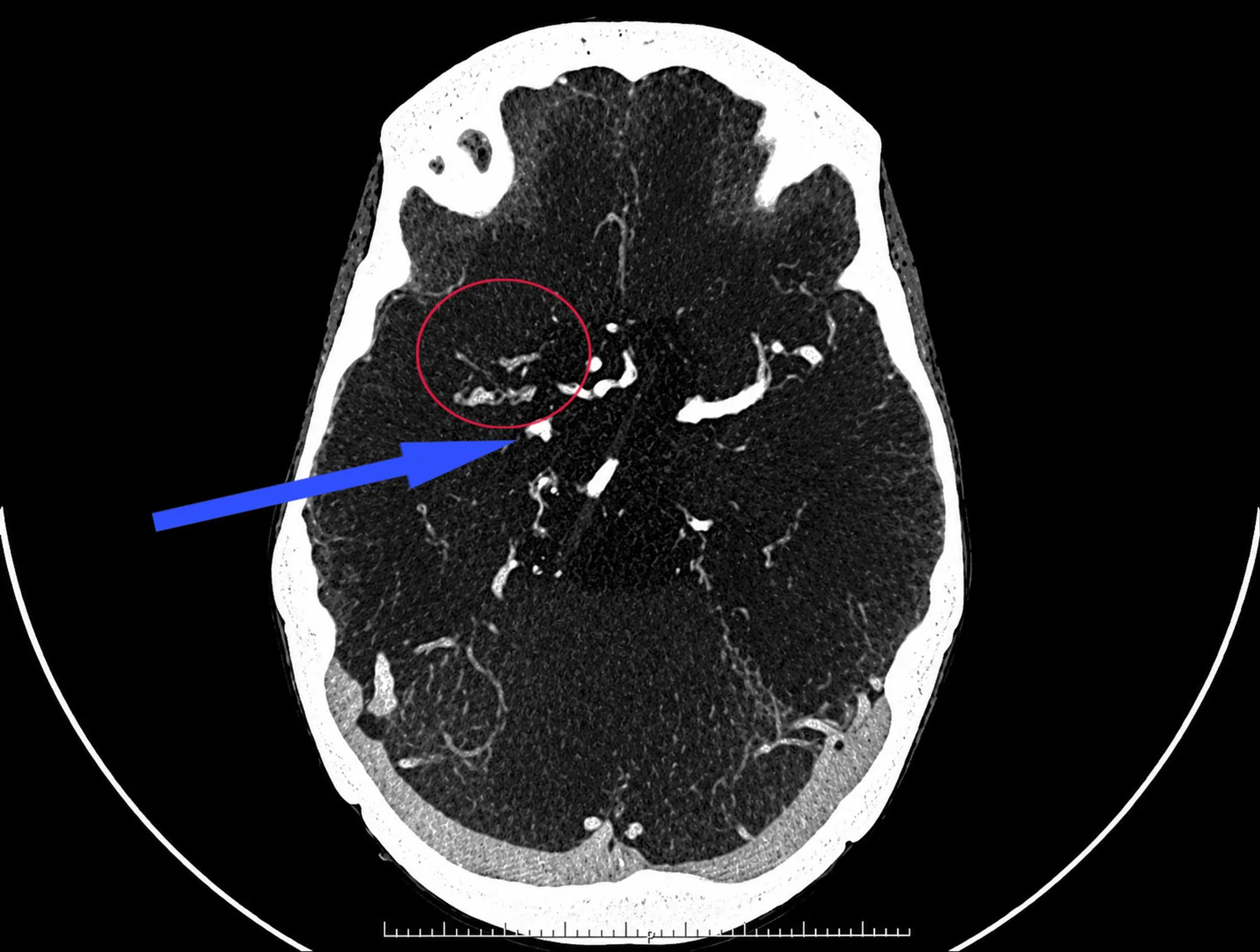
Carotid and cerebral computed tomographic angiography No contrast in the communicating segment of the right internal carotid artery, signifying complex occlusion (blue arrow) with formation of multiple collateral channels (red circle) along the circle of Willis on the right side.

This raised suspicion of vasculitis. A Doppler sonogram of the vessels in the neck was suggestive of a normal study. Carotid and cerebral computed tomographic angiography (CTA) was suggestive of marked diffuse circumferential intimo-medial thickening of the cervical segment of the right internal carotid artery, causing stenosis of approximately 70%. Thick eccentric wall calcification in the supra-clinoid segment and the cavernous segment of the right internal carotid artery (Figure 3), with stenosis of 50% in the petrous segment (Figure 4) and severe stenosis in the supra-clinoid segment (Figure 5), and with complete occlusion in the communicating segment. P1 segment of the right posterior cerebral artery is hypoplastic, and the P2 segment is seen as a continuation og right posterior communicating artery, suggestive of fetal origin of the right posterior communicating artery; however, normal in caliber (Figure 6). Short segment complete occlusion of the M1 segment of the right middle cerebral artery with distal reformation through multiple collateral channels, and reformation of the distal right A1 segment of the anterior cerebral artery through the Circle of Willis (Figure 7) and collaterals, likely suggestive of vasculitis.

She met the American College of Rheumatology (ACR) diagnostic criteria based on age of onset of 40 years or younger, claudication of the extremities, carotid bruits, and angiographic features. Therefore, based on the clinical and angiographic findings, the diagnosis of TA type I was made. Subsequently, the recommended treatment of methylprednisolone was started with an initial dose of 40 mg per day, tab methotrexate 7.5 mg once a week, and tab folic acid 5 mg once daily, six out of seven days. Physiotherapy was initiated on the second day of admission. She was discharged from the hospital on the seventh day of admission in a hemodynamically and clinically stable condition. At the time of discharge, her stroke disability modified Rankin Scale was 1/6. At follow-up visits at 15 days, one month, and three months, the patient's clinical condition was stable, and she had no new clinical signs or symptoms. 

## Discussion

TA is a chronic idiopathic granulomatous large-vessel vasculitis predominantly affecting the aorta and its primary branches. It commonly affects young women of Asian descent and usually manifests during the second and third decades of life [5]. Neurological manifestations are reported in approximately 10-20% of patients and occur secondary to stenosis, occlusion, embolism, or severe hypoperfusion involving extracranial and intracranial vessels [6]. Although constitutional symptoms and diminished peripheral pulses are considered classical features, a cerebrovascular accident may occasionally present initially, thereby making diagnosis difficult in the early stages of disease [7].

The present case highlights an unusual presentation of TA in a young female presenting primarily with ischemic stroke. The patient had no conventional vascular risk factors such as hypertension, diabetes mellitus, smoking, alcohol use, dyslipidemia, oral contraceptive intake, or known cardiac disease. In addition, autoimmune screening was negative, and inflammatory markers were within normal limits except for minimally elevated CRP. Previous studies have demonstrated that inflammatory markers may remain normal in patients with chronic or inactive TA despite ongoing vascular damage [8]. Therefore, ESR and CRP correlate imperfectly with disease activity in TA, and normal values should not exclude the diagnosis in clinically suspicious cases. Accordingly, angiographic progression may occur even in the presence of normal inflammatory markers, highlighting the importance of correlating laboratory findings with vascular imaging.

The patient had intermittent upper limb pain and paresthesia for several months before presentation, likely representing evolving vascular insufficiency. The presence of a carotid bruit on physical examination was an important clinical clue suggestive of large-vessel involvement. Interestingly, all peripheral pulses were palpable with symmetrical blood pressure in both upper and lower limbs. This finding emphasizes that the classical “pulseless disease” phenotype may not always be present, particularly in early or localized disease [9]. In our patient, preserved peripheral pulses and symmetrical blood pressure measurements initially reduced clinical suspicion despite advanced intracranial vascular disease, thereby illustrating that normal pulse examination does not exclude TA.

Doppler ultrasonography of the neck vessels was unremarkable because the inflammatory changes predominantly involved the intracranial internal carotid artery and distal cerebral vessels, which are not adequately evaluated by cervical Doppler ultrasonography. Neuroimaging as well as vascular imaging therefore provided superior assessment of the extent and distribution of vascular involvement and thus were instrumental in establishing the diagnosis. MRI brain demonstrated chronic infarcts and collateral vessel formation, suggesting longstanding progressive cerebrovascular disease. CTA revealed circumferential intimo-medial thickening involving the cervical and intracranial segments of the right internal carotid artery with severe stenosis and occlusion of the communicating segment, along with occlusion of the right middle cerebral artery and extensive collateralization. Such imaging findings are highly suggestive of TA and help differentiate it from premature atherosclerosis, moyamoya disease, fibromuscular dysplasia, and isolated central nervous system vasculitis [10].

The pathogenesis of ischemic stroke in TA is multifactorial and may result from chronic hypoperfusion, artery-to-artery embolism, thrombosis, or severe stenotic disease affecting major cerebral vessels [11]. Intracranial vessel involvement is less common but has increasingly been recognized with the use of advanced angiographic modalities. The extensive collateral circulation observed in this patient likely represented a compensatory mechanism due to chronic progressive arterial occlusion.

The differential diagnosis included primary angiitis of the central nervous system (PACNS), moyamoya disease, fibromuscular dysplasia, and premature atherosclerosis. PACNS was considered because of the significant intracranial arterial stenosis and ischemic stroke. However, PACNS is primarily restricted to the vessels of the central nervous system. In contrast, our patient had characteristic circumferential arterial wall thickening involving the cervical internal carotid artery in addition to intracranial disease. The presence of a carotid bruit and preceding upper-limb symptoms also suggested vascular involvement beyond the cerebral circulation. Furthermore, the extensive collateral formation and chronic infarcts were consistent with a slowly progressive occlusive vasculopathy. Although normal inflammatory markers may occur in chronic vascular inflammatory disorders, the combination of the patient's young age, clinical vascular symptoms, carotid bruit, extracranial arterial wall thickening, and characteristic angiographic abnormalities favored TA over isolated PACNS. Moyamoya disease typically presents with progressive bilateral terminal internal carotid artery stenosis and characteristic basal collateral vessels. Still, it lacks the concentric arterial wall thickening and systemic large-vessel involvement seen in TA. Fibromuscular dysplasia predominantly affects the renal and extracranial carotid arteries and is characterized by a typical “string-of-beads” appearance rather than inflammatory arterial wall thickening. In contrast, premature atherosclerosis is uncommon in young women without conventional vascular risk factors and usually does not demonstrate diffuse concentric vessel wall thickening. In our patient, the combination of young age, carotid bruit, circumferential arterial wall thickening, and characteristic angiographic involvement of the internal carotid artery favored TA over these alternative diagnoses [6, 9-11].

According to the 1990 American College of Rheumatology criteria, TA can be classified based on age younger than 40 years at disease onset, claudication of the extremities, decreased brachial artery pulses, blood pressure discrepancy, bruits over major arteries, and characteristic angiographic abnormalities [12]. The present patient fulfilled significant criteria, including age below 40 years, presence of carotid bruit, and characteristic angiographic findings involving major vessels.

Management of TA focuses on suppression of vascular inflammation and prevention of ischemic complications. High-dose corticosteroids remain the first-line treatment and are frequently combined with steroid-sparing immunosuppressive agents such as methotrexate, azathioprine, or mycophenolate mofetil [13]. Antiplatelet therapy and neurorehabilitation are essential in patients presenting with stroke. Surgical or endovascular revascularization procedures may be considered in selected patients with severe symptomatic stenosis after adequate control of active inflammation [14]. In our patient, methotrexate was selected as a steroid-sparing immunosuppressive agent because it is recommended as an adjunct to glucocorticoid therapy in TA to facilitate corticosteroid tapering and reduce the risk of disease relapse. Long-term management involves gradual glucocorticoid tapering, continued immunosuppressive therapy, and periodic clinical and vascular imaging surveillance to monitor disease activity and detect vascular progression or relapse [13].

A timeline of clinical presentation, investigations, diagnosis, treatment, and follow-up is provided in Table 2.

**Table 2:** Timeline of clinical presentation, investigations, diagnosis, treatment, and follow-up NCCT: non-contrast computed tomography, MRI: magnetic resonance imaging, CTA: computed tomographic angiography

Time	Event
Three to four months before admission	Intermittent right upper-limb pain and numbness suggestive of upper-limb claudication
Six days before admission	Right-sided upper and lower limb weakness
One day before admission	Dysphagia
Day 1	NCCT head showed chronic infarct
Day 1	Contrast-enhanced MRI brain demonstrated chronic infarcts, intracranial arterial narrowing, and collateral formation
Day 2	Carotid Doppler normal
Day 2	CTA demonstrated diffuse circumferential thickening and severe stenosis of the right internal carotid artery and its branches, consistent with Takayasu's arteritis
Day 2	Methylprednisolone and methotrexate initiated; physiotherapy started
Day 7	Discharged with clinical improvement
15-day follow-up	Stable, no new neurological deficits
One-month follow-up	Clinically stable
Three-month follow-up	Stable without new symptoms

Long-term prognosis depends on disease activity, the extent of vascular involvement, and timely initiation of immunosuppressive therapy. Even after clinical remission, patients require periodic clinical assessment together with serial vascular imaging because vascular progression may occur despite minimal symptoms or normal inflammatory markers [6,13].

This case underlines the importance of considering TA in the differential diagnosis of young-onset ischemic stroke, especially in women without traditional vascular risk factors. Careful vascular examination and timely angiographic evaluation are critical for early diagnosis and prevention of irreversible neurological deficits.

This report has several limitations. As a single case report, it cannot establish causal relationships or define diagnostic patterns for TA presenting with ischemic stroke. Nevertheless, it highlights an atypical presentation that may increase clinical awareness and facilitate earlier diagnosis in similar patients.

## Conclusions

TA is an important yet often underrecognized cause of ischemic stroke in young women. The absence of classical findings, such as absent peripheral pulses or markedly elevated inflammatory markers, may delay diagnosis. Carotid bruit, recurrent transient neurological symptoms, and characteristic angiographic findings of large-vessel stenosis with collateral formation should raise suspicion for TA. Early recognition and prompt initiation of immunosuppressive therapy are crucial in preventing recurrent cerebrovascular events and long-term vascular complications. This case emphasizes the need for heightened clinical awareness of large-vessel vasculitis in young patients presenting with stroke without conventional risk factors.
